# Babassu‐Derived Sachet for *Pereskia aculeata*–Based Oily Sauce: A Bio‐Compostable Single‐Use Packaging Option

**DOI:** 10.1111/1750-3841.70690

**Published:** 2025-11-12

**Authors:** Letícia de Oliveira Gonçalves, Patrícia Marques De Farias, Thalita Ferreira de Freitas, Lilia Zago, Ricardo Felipe Alves Moreira, Bianca Chieregato Maniglia, Ana Elizabeth Cavalcante Fai

**Affiliations:** ^1^ Food and Nutrition Graduate Program Federal University of the State of Rio de Janeiro (UNIRIO) Rio de Janeiro Rio de Janeiro Brazil; ^2^ Sustainable Packaging Institute SPI, Faculty of Life Sciences Albstadt‐Sigmaringen University Sigmaringen Germany; ^3^ Laboratory of Multidisciplinary Practices for Sustainability (LAMPS), Department of Basic and Experimental Nutrition, Institute of Nutrition Rio de Janeiro State University (UERJ), Maracanã Rio de Janeiro Rio de Janeiro Brazil; ^4^ São Carlos Institute of Chemistry (IQSC) University of São Paulo (USP) São Carlos São Paulo Brazil

**Keywords:** babassu cake, bio‐based food package, bio‐compostable sachets, bio‐disintegration

## Abstract

Films were produced from babassu mesocarp flour (BM) and from a composite with BM cake (BMC), a by‐product of oil extraction; both were subjected to alkaline and hydrothermal treatments. The films were tested as sachets for packaging an artisanal ora‐pro‐nóbis pesto. BMC films exhibited higher mechanical strength, with a tensile strength of 2.20 MPa and a Young's modulus of 4.29 MPa. In contrast, BM films reached an elongation of 38.3%. Both films were uniform, dark black in color, provided approximately 98% ultraviolet (UV) shielding, completely biodegraded in soil within 90 days, and were non‐toxic to bean seeds. BMC, in particular, acted as a more effective biofertilizer and promoted germination by 93% with a germination rate index of 13.39 seeds/day. In an aqueous environment, both films decomposed similarly in freshwater (∼6.3% after 14 days), although BMC decomposed more in seawater (5.95%) than BM (0.13%). Antimicrobial testing showed that BM inhibited *Listeria monocytogenes* and *Pseudomonas aeruginosa* at a minimum inhibitory concentration of 6.25 µL/mL, whereas BMC was also effective against *Campylobacter jejuni*, requiring only 3.125 µL/mL to inhibit *L. monocytogenes*. As pesto sachets, the BMC films maintained their microbiological safety and consumer acceptability for 4 days at 4°C and 25°C and showed a high puncture resistance of 27.5 N despite minor pH and color changes. Overall, the BMC sachets proved to be a biocompostable alternative for single‐use packaging of oily sauces, adding value to by‐products in the context of a circular bioeconomy.

## 1 Introduction

The growing global popularity of sachets, single‐use fossil fuel‐based packaging, usually consisting of multilayer structures, is due to their convenience and wide range of applications in various sectors such as cosmetics, food, and pharmaceuticals (Rangel‐Buitrago et al. 2024). One of the main advantages of this packaging system is the provision of pre‐measured portions, which ensure standardized quantities of the product. These applications increase the convenience of packaged food for the consumer and improve the efficiency of food companies and large‐scale food production (Demircan and Velioglu 2025).

However, the environmental impact of single‐use petrochemical plastics, derived from non‐renewable resources, is a critical problem, especially in marine ecosystems. Their fragmentation into micro‐ and nanoplastics allows them to spread throughout the food chain, ultimately reaching humans and threatening both ecosystem integrity and public health (Abrokwah et al. 2022; Bugatti et al. 2023; Petkoska et al. 2021). In response to these challenges, the search for more sustainable food sachets has intensified. Research is focusing on biodegradable polymers to reduce microplastic pollution and on bio‐based polymers to facilitate the transition to renewable raw materials (Velasquez et al. 2025).

In this context, the babassu (*Attalea speciosa*) is a reference for plant extractivism in Brazil. Oil extraction generates large amounts of by‐products (Raposo et al. 2021), such as the mesocarp, an alternative source of starch (∼85%) with notable fiber content (11%), and the cake, a fibrous fraction composed of ∼42% carbohydrates, 29% lipids, 19% proteins, and ∼29% fibers (Ferrari and Soler 2015; Maniglia et al. 2017; Rodrigues et al. 2020). The mesocarp also contains 98.3 mg phenolic compounds per 100 g (Maniglia et al. 2017), and a similar composition is expected for the cake. These properties highlight the ability of the mesocarp to form bio‐based films (Maniglia et al. 2017) and the potential of the cake as a polymer matrix or reinforcing agent (De Farias et al. 2021; Jagadeesh et al. 2021). However, the high fiber content can impair the adhesion of the polymers and prevent the formation of homogeneous films (Jagadeesh et al. 2021). To overcome this limitation, alkaline and thermal treatments are applied to improve fiber–matrix interactions, facilitating the extraction of carbohydrates, lipids, and proteins into the supernatant and resulting in more stable film‐forming solutions (Samir et al. 2022; Ying et al. 2018; Maniglia et al. 2017).

Despite its potential, BM cake (BMC) remains underexplored, and, to the best of our knowledge, there are no studies reporting on its use in the packaging sector, highlighting a clear research gap. Considering its unique composition, the socio‐economic importance of babassu in Brazil, and the urgent need to replace single‐use plastics, the valorization of BMC as a polymer matrix proves to be an innovative strategy in line with the 2030 Agenda.

For the application side, pesto was selected as a model product. Originally from Italy, pesto is the second most popular pasta sauce after tomato sauce. Its greenish color, taste, and texture are determined by the mixture of extra virgin olive oil, Parmesan or Pecorino cheese, oilseeds, garlic, and basil (De Bruno et al. 2022; Zardetto and Barbanti 2020). As a substitute for basil, *Pereskia aculeata*, known as ora‐pro‐nóbis, is an unconventional food plant (UFP) native to Brazil and widespread throughout the country, with a high content of protein (∼25%) and minerals such as calcium, magnesium, manganese, iron and zinc (Lira et al. 2023; Silva et al. 2019).

Therefore, this study develops and characterizes compostable, ultraviolet (UV)‐protective sachets from babassu mesocarp flour (BM) and BMC supernatant for artisanal ora‐pro‐nóbis pesto packaging.

## 2 Methodology

### 2.1 Materials

BM (*A. speciosa*) (moisture: 12.08%; protein: 1.76%; ash: 0.90%; lipid: 0.33%; carbohydrate: 84.57%, of which 56.40% is starch) was supplied by Vem do Xingu (Altamira, PA, Brazil). BMC (moisture: 7.07%; protein: 24.73%; ash: 4.16%; lipid: 15.24%; carbohydrate: 48.81%; multi‐elements [µg/g], as Al: 19.3; Ca: 905; Cu: 30.5; Fe: 170; K: 9707; Mg: 4060; Mn: 322; Na: 50.7; P: 19,859; and Zn: 78.1) was kindly provided by Florestas Brasileiras Ltda (Itapecuru Mirim, Maranhão, Brazil). Glycerol was provided by Chepplier (Rio de Janeiro, Brazil). NaOH was supplied by Dinâmica Química Contemporânea Ltda (São Paulo, Brazil). The extra virgin olive oil, cashew nuts, aged cheese, and ora pro nóbis leaves (*P. aculeata*) were purchased at a local market in Rio de Janeiro.

### 2.2 Preparation of Babassu Films

#### 2.2.1 Preparation and Alkaline Treatment of Babassu Cake

In order to define the methodology of the film‐forming suspensions (FFSs), some preliminary tests were carried out: (i) babassu cake flour (unsieved and sieved with different mesh sizes) in combination with water and glycerol, heated in an autoclave and water bath; (ii) BMC supernatant at different pH values in combination with glycerol, heated in an autoclave and water bath; and (iii) BMC supernatant in combination with BM flour, at different pH values, heated in an autoclave and water bath. This third method was the only one with which homogeneous, crack‐free films could be formed. The alkaline autoclave–heated treatment (pH 12) was chosen due to the aspects of the films obtained in the preliminary testing phase, the results of De Farias et al. (2023) and the ability to bring more polymers into the supernatant (Maniglia et al. 2017).

The BMC was triturated in a blender (Philips Walita, Brazil) and then sieved through a 100‐mesh sieve. A 5% (w/w) BMC suspension was prepared with NaOH solution (1 N) to increase the native pH (∼6) to pH 12 (by adding 1 mL of NaOH solution per 20 g of supernatant). The suspension was allowed to stand for 12 h under refrigeration and then filtered using a fabric filter. The supernatant of BMC filtered at pH 12 (cake supernatant) (moisture: 31.71%; protein: 19.19%; ash: 0.64%; lipid: 3.08%; carbohydrate: 45.39%; and 10 g/L soluble solids) was obtained for the preparation of the film suspension according to the method of De Farias et al. (2023). In contrast to the BMC, the supernatant was selected for its ability to form a film, which is determined by its lower fiber content.

#### 2.2.2 Preparation of Film‐Forming Suspension by Hydrothermal Treatment

The FFSs were based on a polymeric matrix of BM flour (Maniglia et al. 2017). Two FFSs were prepared as follows: (FFS 1) 4 g BM flour in 96 g water adjusted to pH 12 with NaOH and (FFS 2) 4 g BM flour in 96 g cake supernatant. In both cases, a 30‐min hydrothermal treatment in an autoclave at 121°C was performed. Glycerol (30 g/100 g BM flour) was then added according to the method of Santos et al. (2023). The FFS was poured onto acrylic sheets (0.28 g/cm^2^) and dried in an oven (Marconi, Brazil) at 35°C for 18 h or until completely dry. The dried films were conditioned in a desiccator at 25°C and 53% relative humidity (RH) for 48 h before analysis. FFS 1 and 2 yielded a BM film and a BM film with cake (BMC film).

### 2.3 Characterization of Babassu‐Based Films

#### 2.3.1 Mechanical Properties

The mechanical properties were determined according to American Society for Testing and Materials (ASTM) method D882‐12 (ASTM 2012), whereby each sample (70 × 25 mm^2^) was tested in quintuplicate on a TX‐700 texturometer (Lamy Rheology, France) (Matheus et al. 2021).

#### 2.3.2 Visual Aspect, Light Transmission, and Color Stability

The visual aspect of the films was recorded with the camera of an Iphone 11 (Apple, USA, 4000 × 3000 pixels). The light transmittance of the films was analyzed in the UV and visible range (200–800 nm) using a UV–Vis spectrophotometer (UV‐2700 Shimadzu, Japan) (Nouraddini et al. 2018). The percentages of UV blockage (*S*_UV_) and visible light (*S*_Vis_) were determined according to following equations adopted from Silva et al. (2024):

(1) $$S_{\mathrm{UV}}\left(\%\right)=1-T_{\mathrm{UV}}=1-\frac{\sum \frac{400}{200}T\lambda \times \Delta \lambda }{\sum \frac{400}{200}\Delta \lambda }\times 100$$

(2) $$S_{\mathrm{Vis}}\left(\%\right)=1-T_{\mathrm{Vis}}=1-\frac{\sum \frac{800}{400\;}T\lambda \times \Delta \lambda }{\sum \frac{800}{400}\Delta \lambda }\times 100$$

where *T*_UV_ and *T*_Vis_ represent the average percentage transmittance of UV and visible light, respectively, calculated using the spectral transmittance (*Tλ*) and the wavelength interval (Δ*λ*).

The color stability of the films was examined using a colorimeter (3nh, Colorimeter Spectrometer Y53020, China) in triplicate under two different conditions: refrigeration (4°C ± 2°C) and room temperature (25°C ± 2°C). The samples were exposed to light and darkness after 0, 3, 7, 14, 21, and 28 days. The method was adopted from Mohammadalinejhad et al. (2020). The color coordinates L*, a*, and b* of the CIE color scale were analyzed with a colorimeter using a D65 reference illuminant, 10° observer and calibrated with a standard white reflector plate (L* 94.98, a* −0.22, and b* 0.37). The colorimetric changes were compared to the samples at baseline to calculate the whiteness index (Equation 3):

(3) $$\mathrm{Whiteness}\;\mathrm{index}:100-\sqrt{{\left(100-L\right)}^{2}+a^{2}+b^{2}}$$

where *L, a*, and *b* are the parameters at the checkpoint.

#### 2.3.3 Bio‐Disintegration and Phytotoxicity Tests

The bio‐disintegration of the films in the soil under home composting conditions was tested according to the method of Aldas et al. (2021). Twenty‐one samples measuring 3 cm × 3 cm were prepared from each film, the control film (cellulose) and the PVC. The samples were wrapped in a fine plastic net and buried in a glass container with 2.5 kg of garden soil (2:1 soil, humus), with 200 mL of water poured into each container daily. Samples were collected on Days 7, 15, 30, 45, 60, 75, and 90 for visual assessment. Each sample was collected in triplicate at the seven control points. The bio‐disintegration of the films in freshwater (pH = 8.2) and seawater (pH = 7.1) was also studied over 2 weeks using adapted methods according to Pereira et al. (2021) and Filipini et al. (2020).

Phytotoxicity analysis was continued with the final soil from the bio‐disintegration test samples and for the virgin mixed soil. One hundred bean seeds were planted in each soil type, irrigated once a day with 200 mL of water and kept at 25°C ± 2°C. Their growth was visually assessed for 14 days and the number of germinated seeds, initial germination time and maximum leaf of each seedling were quantified according to Díaz‐Díaz et al. (2023) with modifications. Germination rate (%*G*), mean germination time (MGT), and germination speed index (GSI) were calculated (Equations (4), (5), (6)) as described by Rêgo et al. (2017):

(4) $$\%G=\frac{\mathrm{number}\;\mathrm{of}\;\mathrm{germinated}\;\mathrm{seeds}}{\mathrm{total}\;\mathrm{seeds}}\times 100$$

(5) $$\begin{array}{ccc} & & \mathrm{MGT}\\ & & =\frac{\sum \mathrm{number}\;\mathrm{of}\;\mathrm{germinated}\;\mathrm{seeds}\times \mathrm{incubation}\;\mathrm{time}\;\mathrm{in}\;\mathrm{days}}{\sum \mathrm{germinated}\;\mathrm{seeds}\;\mathrm{per}\;\mathrm{day}} \end{array}$$

(6) $$\begin{array}{ccc} & & \mathrm{GSI}\left(\frac{\mathrm{seed}}{\mathrm{day}}\right)\\ & & =\frac{\sum \mathrm{number}\;\mathrm{of}\;\mathrm{seeds}\;\mathrm{germinated}\;\mathrm{per}\;\mathrm{day}}{\mathrm{number}\;\mathrm{of}\;\mathrm{days}\;\mathrm{elapsed}\;\mathrm{since}\;\mathrm{sowing}\;\mathrm{and}\;\mathrm{germination}} \end{array}$$

#### 2.3.4 Antimicrobial Activity, Minimum Inhibitory Concentration (MIC), and Minimum Bactericidal Concentration (MBC)

The antimicrobial properties of the films were evaluated in triplicate using the agar diffusion method (Li et al. 2022). The microorganisms used in this test were *Escherichia coli, Salmonella* sp., *Staphylococcus aureus, Bacillus cereus*, *Pseudomonas aeruginosa, Campylobacter jejuni*, and *Listeria monocytogenes*.

MICs were determined in 96‐well microplates by diluting 100 µL of each film solution in 100 µL of brain–heart infusion broth (BHI) containing the respective bacteria against which each film showed antimicrobial activity. Bacteria were diluted in BHI with McFarland turbidity standard and used for growth monitoring. The sterility control was performed with BHI broth and 0.85% saline, whereas the toxicity control was performed with NaOH solution (5%), the medium used to prepare BM and BMC. BM and BMC at native pH were used as controls (C‐BM and C‐BMC, respectively) without pH adjustment. The microplates were incubated at 37°C for 24 h. MBCs were determined by culturing 20 µL of all inhibitory concentrations on Mueller–Hinton agar and incubating the plates at 37°C for 24 h (Santos‐Filho et al. 2019).

#### 2.3.5 Screening Assays of Migration Compounds

The protocols of EU 2016/1416 were applied to a screening analysis to identify potential migrant compounds by immersing each 30 mm × 20 mm film in 10 mL of two simulants: Ethanol 95% (v/v) as a fatty medium and acetic acid 3% (v/v) as an aqueous medium. The samples immersed in the simulants were stored in a temperature‐controlled environment (20°C) and after 10 days the supernatant of each film in each type of simulant was analyzed in a spectrophotometer at wavelengths from 200 to 800 nm (Matheus et al. 2024).

#### 2.3.6 Thermal Properties and Heat Seal Strength

A TA 2010 DSC instrument controlled by a TA5000 module (TA Instruments, USA) coupled with a cryoscopy cooling accessory was used to determine the thermal properties of the films (De Farias et al. 2023). Each 5‐mg dry sample was placed in the aluminum containers and then sealed. All measurements were performed under a nitrogen atmosphere (45 mL/min). Universal Analysis 2000 software (TA Instruments, New Castle, DE) was used with a heating rate of 10°C/min from −150°C to 150°C.

In addition, a TGA‐Q500 thermogravimetric analyzer (TA Instruments, EUA) was used. For this purpose, approximately 10 mg of each sample was carefully measured and placed in platinum dishes. The investigated temperature range was 10–700°C; the heating rate was 10°C/min under a nitrogen atmosphere. The continuous change in mass with temperature was recorded (De Farias et al. 2021).

The seal strength of the films was tested with a texturometer by placing two 45 × 10 mm^2^ pieces of film on top of each other and sealing them at a temperature of approximately 70°C for 5 s (RG‐300L Registron, Brazil). For the test, all sealed films were stored in triplicate at 53% RH in a chamber with controlled RH for at least 48 h. The strength of the seal was determined by placing the ends of the sealed film perpendicular to the test direction with a distance of 50 mm between the clamps and a loading speed of 1.5 mm/s (Alves et al. 2022). The resistance of the sealed material was calculated using the following equation:

(7) $$\mathrm{Seal}\;\mathrm{strength}=\frac{\mathrm{Peak}\;\mathrm{force}\;\left(N\right)}{\mathrm{Film}\;\mathrm{width}\;\left(\mathrm{mm}\right)}$$

### 2.4 Film Application as Food Packaging

#### 2.4.1 Preparation of Pesto Sauce With UFP

The pesto sauce developed was prepared with the following ingredients: 130 g extra virgin olive oil, 54 g roasted and salted cashew nuts, 37 g ora pro nóbis leaves, 4 g garlic, and 50 g mature cheese. In the first steps, the ora pro nóbis leaves were cleaned and disinfected with a chlorine solution and the cheese was grated. All ingredients, except the UFP leaves, were blended in a food processor for about 1 min. Then the UFP was added and blended for about 30 s, resulting in 250 g of pesto sauce.

#### 2.4.2 Packaged Pesto Sauce as a Real System Model

The BMC film was used to produce sachets for packaging the pesto sauce. Each sachet, measuring 50 × 60 mm^2^, contained 5 g of pesto. The sachets with the sauce were stored in the refrigerator (4°C ± 2°C) and at room temperature (25°C ± 2°C) and microbiologically tested for *Salmonella* sp., thermotolerant coliforms, molds, and yeasts (Brasil, Agência Nacional de Vigilância Sanitária (ANVISA) 2022). In addition, the peroxide index, pH and colorimetric parameters were determined according to the method described by Glicerina et al. (2023). All analyzes were carried out in triplicate on Days 0, 2, and 4.

#### 2.4.3 Sensory Analysis of Packaged Pesto Sauce

The study was reviewed and approved by the Research Ethics Committee of the *Hospital Universitário Pedro Ernesto* of the Rio de Janeiro State University (CAAE 79549917.4.0000.5259, report 2.694.935), and informed consent was obtained from each subject before participation in the study. Subsequently, 124 untrained panelists were asked to evaluate the sensory parameters of the sauce 2 days after the sachets were filled. The evaluation was based on a 9‐point hedonic scale and tests to predict purchase intention (Instituto Adolf Lutz [IAL] 2008). The word clouds were created with WordArt.

#### 2.4.4 Puncture Test of the Sachets With Packaged Pesto

All analyzes were performed in triplicate on Days 0, 2, and 4 of the packaged pesto. To determine the puncture force, the texturometer was used, in which the packages were fixed with an opening of 30 mm diameter. The force to be transmitted through a 7 mm spherical probe at a speed of 0.8 mm/s was determined using a method adopted from Aparicio‐Fernández et al. (2018). The mechanical test was performed in triplicate at each test point for both samples.

### 2.5 Statistical Analysis

The test data were processed using analysis of variance and Tukey's test, with statistical significance set at the 95% level. The analyzes were performed using the statistical software package Statistica 7.0.

## 3 Results and Discussion

### 3.1 Mechanical Properties, Visual Appearance, Light Transmission, and Color Stability

**FIGURE 1:**
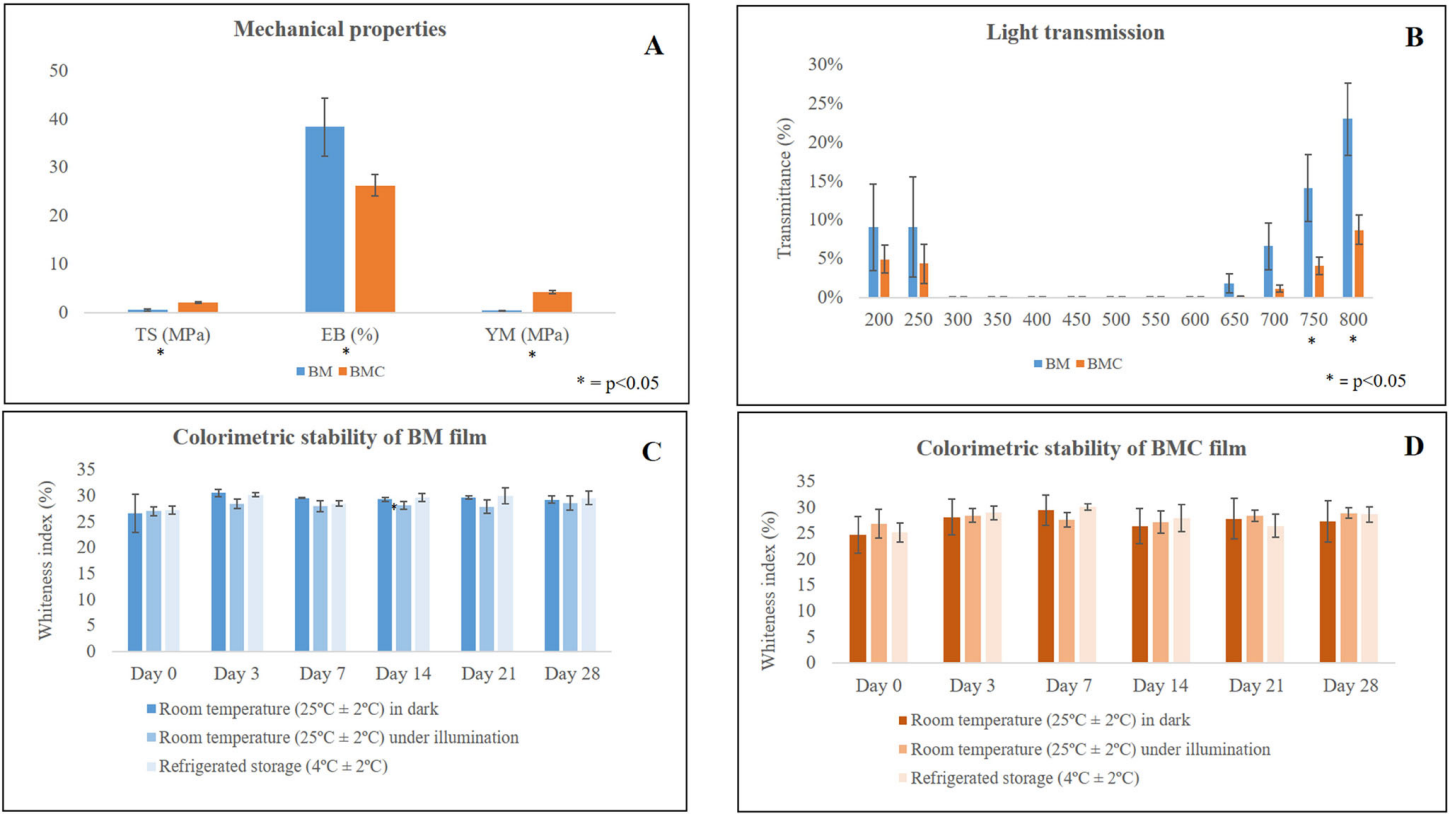
Mechanical properties (A), light transmission (B), and colorimetric stability (C) and (D) of BM and BMC films. BM, babassu mesocarp film; BMC, babassu mesocarp film with cake.

The mechanical properties of the films are shown in Figure 1. The BMC film showed a higher tensile strength (TS: 2.20 ± 0.14 MPa) and a higher Young's modulus (YM: 4.29 ± 0.32 MPa) compared to the BM film (TS: 0.59 ± 0.23 MPa and YM: 0.43 ± 0.12 MPa, respectively). Conversely, the BM film showed greater elongation (38.38% ± 5.99%) than the BMC film (26.37% ± 2.28%). These differences can be attributed to the different compositions of the polymers, especially the fibers that act as fillers in the matrix, resulting in lower mobility and elasticity of the polymer chains and a simultaneous increase in YM and TS (Guo et al. 2018).

The alkaline treatment effectively removes and dissolves some of the hemicellulose and lignin (Guo et al. 2018). However, residual fibers remain attached to the matrix, as can be seen in the BMC film, which contains a significant proportion of untreated fibers (Gasparini et al. 2015). In contrast, this phenomenon is less pronounced in the BM film, which consists mainly of starch (Maniglia et al. 2017).

**FIGURE 2:**
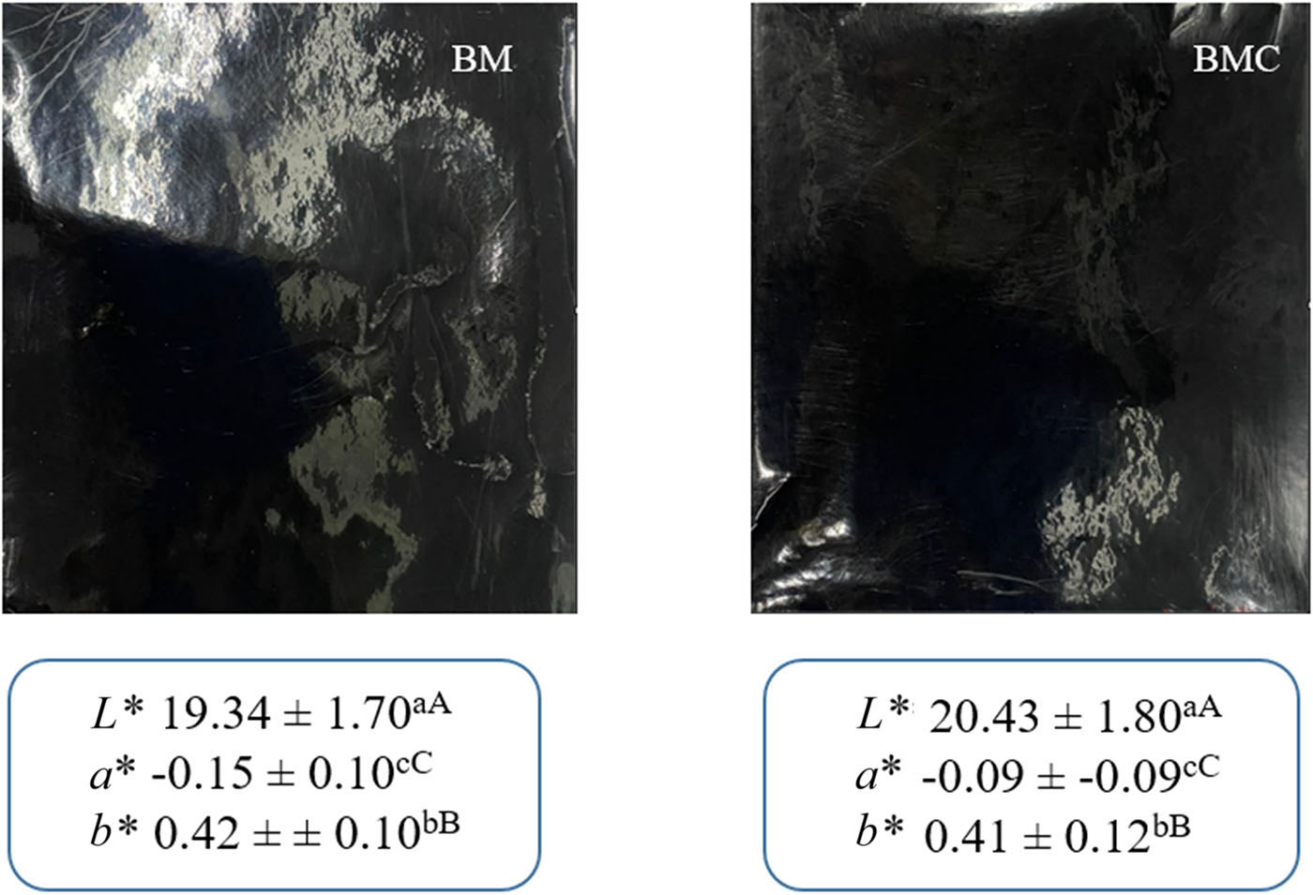
The visual appearance of BM and BMC films. Different superscript letters indicate statistical significance (*p* < 0.05). Lowercase letters are used to compare parameters of the same film, and uppercase letters are used to compare parameters of different films. BM, babassu mesocarp film; BMC, babassu mesocarp film with cake.

Both films had a uniform, non‐cracking surface and a dark black color (Figure 2). However, BMC had a slightly rougher surface than BM due to its fibrous content and the pronounced granulometry of BMC compared to mesocarp flour, which can form tiny air pores during the production process.

The light transmission of BM and BMC films is shown in Figure 1. The combination of cake supernatant and BM flour, which contains vivid pigments, results in a darker film solution. In addition, the presence of carbohydrates and proteins, in these matrices, combined with alkaline and hydrothermal treatments, may possible trigger caramelization and Maillard reactions (Nogueira and Martins 2019; Tan et al. 2021; Sioriki et al. 2021), resulting in darkening of the film and reduced light transmission.

In the UV range, no significant differences were found between the films, with BM and BMC blocking 98.42% and 98.59% respectively. However, at 750 and 800 nm, BMC had lower light transmittance (4.05 ± 0.01 and 8.74 ± 0.02, respectively) than BM (14.14 ± 0.04 and 23.02 ± 0.05, respectively), corresponding to visible light blockage of 98.36% and 94.37%, respectively. One hypothesis is that the anti‐UV properties are due to the presence of lignin in the mesocarp and, possibly, in the cake supernatant (Silva et al. 2008, 2023). This property is related to phenolic hydroxyl chromophores, stacked aromatic π–π rings, and conjugated double bonds, that can absorb UV radiation (Zhang and Jiang 2023). Hemicellulose, which may be dissolved in the supernatant under alkaline conditions (Silva et al. 2008), is formed by the expansion of biomass and the dissolution of lignin binding (Lima et al. 2014). This increases the accessibility, of carbohydrates and promotes caramelization reactions, which are expected to affect the color of the film and UV blocking ability (Worku et al. 2024). In addition, heat treatment is thought to contribute to darkening by producing colored compounds from the degradation and oxidation of hemicellulose, lignin, and other polysaccharides, that release quinones and methylquinones (Akkus and Budakçı 2020).

The dark coloration, low light transmission, and blocking properties of the films indicate an excellent barrier potential for UV light. This could be beneficial for foods that are normally already packaged in dark material, such as those with volatile compounds or rich in fats, and favor more sustainable practices.

Color stability is a decisive factor in the selection of suitable packaging films. As shown in Figure 1, no statistically significant differences were found between BM and BMC films under the same storage conditions at different times or between films stored under different conditions at the same time.

In contrast to the results of Zhang et al. (2019), who reported color changes in indicator films under different storage conditions, the films in this study showed no statistically significant differences between refrigerated and non‐refrigerated storage. This suggests that both BM and BMC films have high colorimetric stability and effectively retain their dark pigments, which may contribute to their observed anti‐UV properties.

### 3.2 Bio‐Disintegration and Phytotoxicity Tests

**FIGURE 3:**
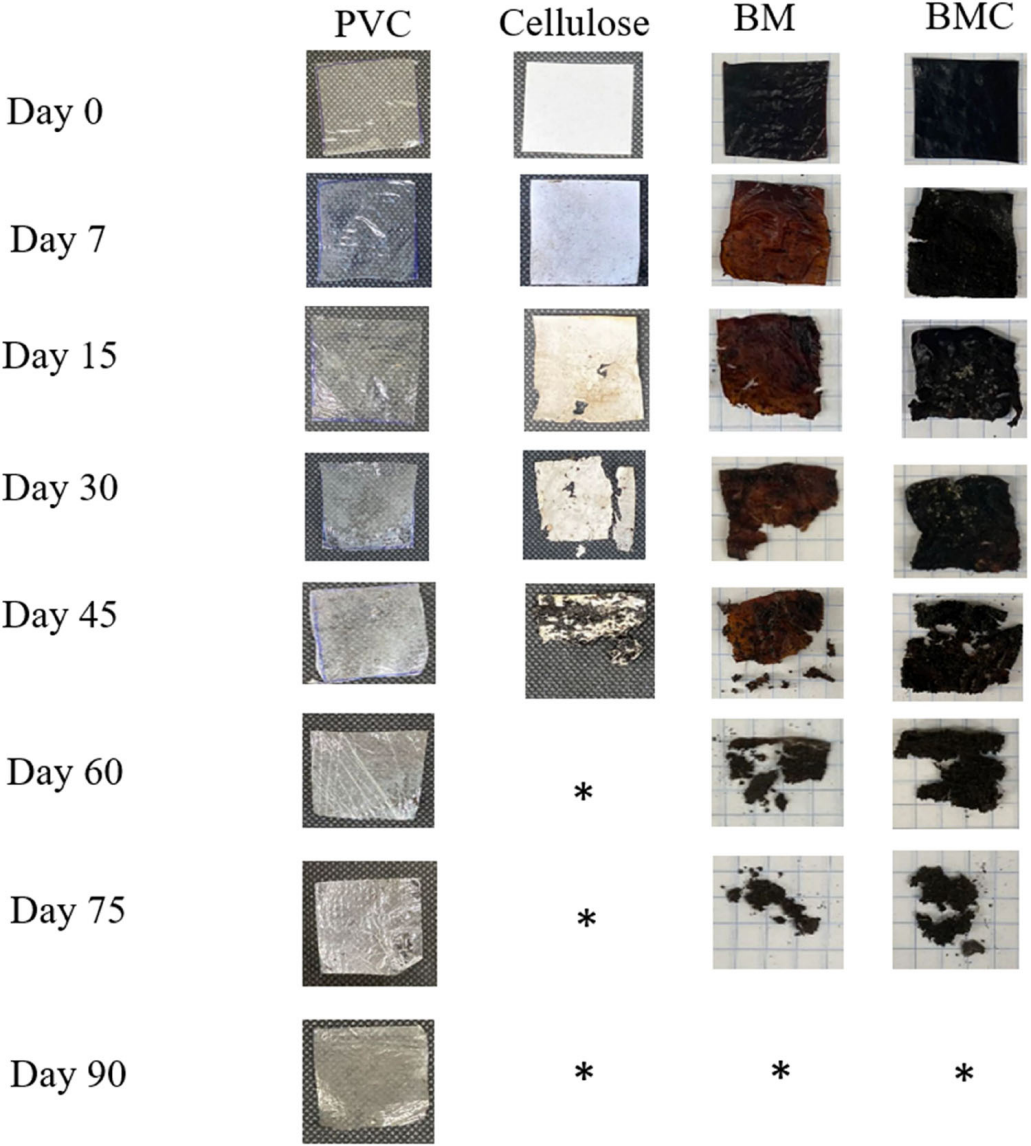
Disintegration rate of BM and BMC films, and controls (PVC and cellulose) buried in soil for 90 days. BM, babassu mesocarp film; BMC, babassu mesocarp film with cake.

Figure 3 illustrates the bio‐disintegration of the films in the soil as a function of the incubation time. On Day 7, the first signs of disintegration, including color change, cracking and/or roughness, were observed in the BM and BMC films. However, a noticeable difference in cellulose was not observed until Day 15. After 15 days, BM was found to have the fastest overall disintegration after cellulose.

During irrigation, the films in the soil gradually disintegrate due to water infiltration and microbial activity causing swelling and progressive mass loss (Dirpan et al. 2023). Chemical and thermal treatments significantly accelerate disintegration. These processes can hydrolyze the bonds and change the chemical structures of the polymers, transforming them into simpler, more hydrophilic compounds that are more susceptible to microbial activity and proliferation (Maniglia et al. 2017; Momen et al. 2021; Zborowska et al. 2022).

Films of arrowroot starch were completely disintegrated in 7 days (Abdillah and Charles 2021). Pure starch degrades faster due to its high affinity for water, which promotes swelling and hydrolysis of the polymer and improves microbial accessibility (Sanyang et al. 2015).

The presence of additional polymers besides carbohydrates increases the structural complexity of films and can delay their disintegration. Medina‐Jaramillo et al. (2017) found that films made from cassava starch in combination with plant extract already showed signs of disintegration on Day 6. This was also observed in the films in this study, as the films containing only BM flour (consisting of 84.93% carbohydrates) showed faster signs of disintegration than the films with cake supernatant.

The disintegration of BM and BMC films was investigated in seawater and freshwater. In seawater, BMC showed a significantly higher disintegration rate (5.95% ± 0.10%) than BM (0.13% ± 0.04%), probably due to Na⁺‐ and Cl^−^ ions interacting with heterogeneous regions in the BMC structure and causing local cracking (Weisbrich et al. 2024). However, no significant difference between the two types of films was observed in freshwater (BM: 6.35%; BMC: 6.84%).

No significant difference was found between the BMC film in seawater and in freshwater. However, the BMC film disintegrated more in freshwater (6.35% ± 1.56%) than in seawater (0.13% ± 0.04%). These results indicate that the formulation of the film directly influences its disintegration behavior depending on the medium. This contradicts the general expectation that all films degrade faster in freshwater, which usually has a higher pH and a higher microbial load (Dirpan et al. 2023).

Although the films gave off color in both types of water, this does not necessarily indicate decay, as the dark hydrophilic pigments readily dissolve on contact with water (Tan et al. 2021). However, the presence of odor and turbidity indicates increased microbial activity and progression of the disintegration process. Overall, disintegration occurred faster in soil than in water, probably due to higher microbial density and higher temperatures (Dirpan et al. 2023).

After the BM and BMC films had disintegrated in the soil, the phytotoxicity parameters were evaluated (Table 1).

**TABLE 1:** Germination rate (%*G*), mean germination time (MGT), and germination speed index (GSI) of bean seeds in different soils.

Soil condition	%*G*	MGT	GSI (seeds/day)
Virgin soil	83.0^b^	8.84^c^	8.51^d^
Soil with cellulose disintegrated	73.0^c^	9.04^a^	9.75^c^
Soil with BM film disintegrated	83.0^b^	8.93^b^	9.86^b^
Soil with BMC film disintegrated	93.0^a^	7.45^d^	13.39^a^

*Note*: Values with different letters are significantly different according to Tukey's test (*p* < 0.05).

Abbreviations: BM, babassu mesocarp film; BMC, babassu mesocarp film with cake.

The results show that the disintegrated films and the cellulose as a control are not toxic to the soil and have a positive effect on seed germination and seedling development. The soil with the disintegrated BMC film showed the most favorable parameters, including the highest germination rate (93%), lower MGT (7.45), and faster GSI (13.39 seeds/day) compared to the virgin and other soils. These results emphasize the positive and stimulating effect of the cake supernatant. The soil with BM film provided the second most significant results, as its germination rate (9.86 seeds/day) was also faster than that of the unaltered soil.

The good performance of seeds in soils containing BMC and BM films was attributed to the enrichment of the soil by the disintegration of the biopolymers. This effect can be attributed to the starch content in the buried materials, which is an optimal carbon source that supports seed germination and seedling growth (Reichert et al. 2022). In addition to carbohydrates, micronutrients, such as phosphorus, potassium, calcium, magnesium, and iron, also play an important role in seed development (Tripathi et al. 2014).

In addition to micronutrients, BMC contains a considerable amount of proteins (Gasparini et al. 2015), which is also reflected in the BMC film (19.19%), which serves as a source of nitrogen and supports improved germination and accelerated seedling growth (Tripathi et al. 2014), as shown in Tables 1 and 2.

**TABLE 2:** Visual appearance and sizes of roots, stems, and leaves of seedlings grown in different soils.

Soil condition	Root (cm)	Stem (cm)	Leaf (cm)	Total (cm)	Visual aspect
Virgin soil	3.00 ± 0.70^b^	13.74 ± 0.54^b^	2.04 ± 0.34^a,b^	19.38 ± 0.73^c^	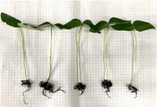
Cellulose	10.52 ± 1.98^a,b^	13.92 ± 1.60^b^	1.30 ± 0.33^b,c^	25.74 ± 2.98^a,b^	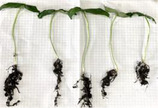
BM film	14.06 ± 4.09^a^	15.58 ± 1.16^b^	1.08 ± 0.17^b,c^	30.72 ± 4.13^a^	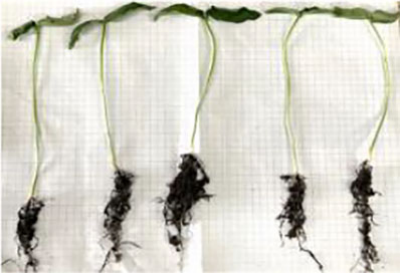
BMC film	5.68 ± 1.76^b^	18.58 ± 0.98^a^	1.50 ± 0.42^b^	26.72 ± 2.69^a,b^	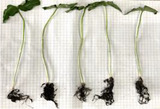

*Note*: Values with different letters in the same column are significantly different according to Tukey's test (*p* < 0.05).

Abbreviations: BM film, babassu mesocarp film; BMC film, babassu mesocarp film with cake.

Variations in soil nutrient profiles led to different developmental responses in seedling components, including roots, stems, and leaves. Increased root growth was observed in soils containing a dissolved BM film or cellulose, whereas stem development was more pronounced in soils with a residual BMC film. These results are consistent with Song et al. (2017), who reported that higher nutrient availability promotes seedling germination and growth.

### 3.3 Antimicrobial Activity by Halo Inhibition Assay, MIC and MBC

**FIGURE 4:**
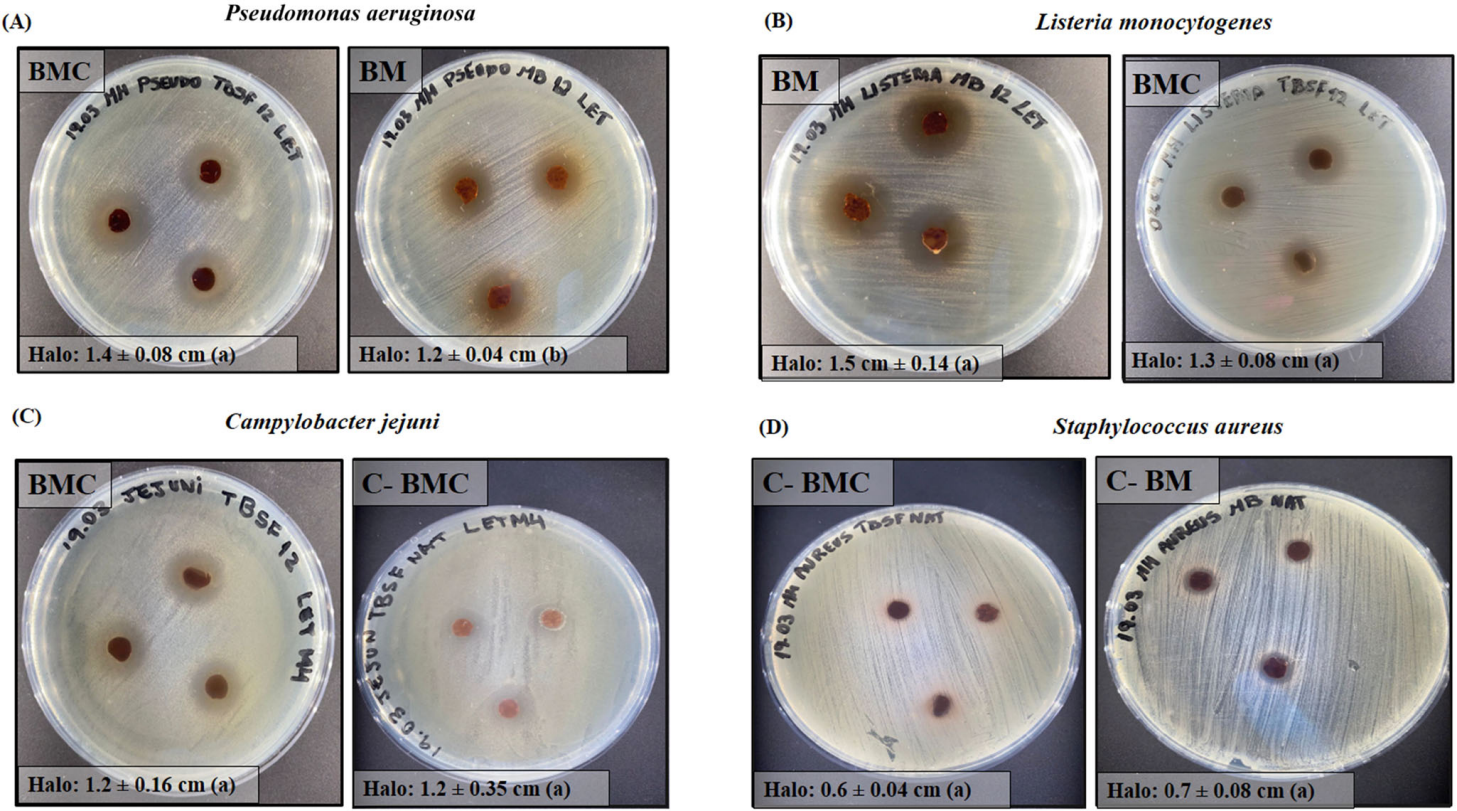
Antimicrobial effect of controls, BM and BMC films against *Pseudomonas aeruginosa* (A)*, Listeria monocytogenes* (B), *Campylobacter jejuni* (C), and *Staphylococcus aureus* (D). Values with different letters for the same microorganism are significantly different according to Tukey's test (*p* < 0.05). BM, babassu mesocarp film; BMC, babassu mesocarp film with cake; C‐BM, control of babassu mesocarp film; C‐BMC, control of babassu mesocarp film with cake.

The results of antimicrobial efficacy are shown in Table 3 and Figure 4. C‐BMC showed antimicrobial activity against *C. jejuni* (12 ± 0.35 mm) and *S. aureus* (6 ± 0.04 mm), whereas C‐BM showed antimicrobial properties only against *S. aureus* (7 ± 0.08 mm). According to Mirres et al. (2024), BM exhibits antimicrobial activity probably due to its content of bioactive compounds, especially polyphenols and phenols. On the basis of this assumption, this could be the case for both the control samples and the films, as they were produced with BM. The BMC film could have enhanced antimicrobial activity compared to BM, possibly due to a synergistic effect between the mesocarp and the cake supernatant, which could also contain bioactive compounds.

**TABLE 3:** Antimicrobial activity, minimum inhibitory concentration (MIC), and minimum bactericidal concentration (MBC) of the films against foodborne pathogens.

Microorganism	Inhibition halo diameter (mm)				MIC (µL/mL)							MBC (µL/mL)
	C‐BM	BM film	C‐BMC	BMC film	C‐BM	BM film	C‐BMC	BMC film	C‐BM	BM film	C‐BMC	BMC Film
*Campylobacter jejuni*	—	—	12 ± 0.35^a^	12.0 ± 0.16^a^	—	—	25.0^b^	6.25^a^	—	—	50.0^b^	50.0^a^
*Pseudomonas aeruginosa*	—	12.0 ± 0.04^b^	—	14.0 ± 0.08^a^	—	6.25^a^	—	6.25^a^	—	50.0^a^	—	50.0^a^
*Listeria monocytogenes*	—	15.0 ± 0.14^a^	—	13.0 ± 0.08^a^	—	6.25^a^	—	3.125^b^	—	50.0^a^	—	50.0^a^
*Staphylococcus aureus*	7.0 ± 0.08^a^	—	6 ± 0.04^b^	—	25.0^a^	—	50.0^a^	—	50.0^a^	—	100.0^a^	—

*Note*: Different letters within each column, for each type of test, indicate significant differences according to Tukey's test (*p* < 0.05).

Abbreviations: BM, babassu mesocarp film; BMC, babassu mesocarp film with cake; C‐BM, control of babassu mesocarp film; C‐BMC, control of babassu mesocarp film with cake.

The BMC film showed antimicrobial activity against a broader spectrum of microorganisms, including *P. aeruginosa*, *L. monocytogenes*, and *C. jejuni*, compared to the BM film. This could possibly be due to the presence of melanoidins, which enhance antimicrobial activity (Diaz‐Morales et al. 2022; Lobiuc et al. 2023).

Microbial susceptibility is assessed using the following parameters: not sensitive for diameters <8 mm, sensitive for diameters 9–14 mm, very sensitive for diameters 15–19 mm, and extremely sensitive for diameters >20 mm (Matheus et al. 2021). Therefore, *C. jejuni* (12 ± 0.16 mm), *L. monocytogenes* (13 ± 0.08 mm), and *P. aeruginosa* (14 ± 0.08 mm) were sensitive to BMC films, whereas *L. monocytogenes* (15 ± 0.14 mm) and *P. aeruginosa* (12 ± 0.04 mm) were highly sensitive and sensitive to BM films, respectively. None of the films showed antimicrobial activity against the other microorganisms tested (*E. coli*, *Salmonella* sp., *S. aureus*, and *B. cereus*). In another study, the ethanolic extract of BM flour showed antimicrobial activity against *S. aureus* (15–18.5 mm), methicillin‐resistant *S. aureus* (MRSA) (15.3–17.4 mm), and *E. faecalis* (12.4–14.4 mm). However, no inhibitory effect against *P. aeruginosa* and *E. coli* was observed (Barroqueiro et al. 2016).

The shift in the microbial inhibition profile of the BM film compared to the C‐BM film could be due to the chemical modification of the compounds in an alkaline environment, which affects the molecular structure and antimicrobial mechanisms of the compounds. Furthermore, the BMC film could contain additional antimicrobial agents, such as melanoidins, which could maintain activity against the microorganisms inhibited by C‐BMC films and thus broaden the antimicrobial spectrum (Diaz‐Morales et al. 2022; Lobiuc et al. 2023; Pasquet et al. 2024).

The MIC and MBC of the films were described in Table 3. C‐BM and C‐BMC had an MIC of 25 and 50 µL/mL against *S. aureus*. C‐BMC presented an MIC of 25 µL/mL against *C. jejuni*. Inhibition of *L. monocytogenes* was achieved with the BMC film at a lower MIC compared to the other microorganisms. At a concentration of 50 µL/mL, no bacterial growth was observed with either film. The positive control (5% NaOH) showed bactericidal activity against all microorganisms tested, which may have contributed to the superior inhibitory and bactericidal activities observed with the BMC and BM films.

A study conducted with Juçara bark extract showed that concentrations of 10 and 20 µL/mL were able to inhibit *L. monocytogenes* and *P. aeruginosa*, respectively (Garcia et al. 2019). However, these concentrations were less effective against these microorganisms than BM and BMC films. In contrast, the ethanolic extract of Camu‐camu bark had an MIC of 5 µL/mL against both *L. monocytogenes* and *P. aeruginosa* (Conceição et al. 2020). It showed a lower sensitivity to *L. monocytogenes* compared to the BMC film and a higher sensitivity to *P. aeruginosa*, also within the same formulation. As observed in the two studies, the extracts showed bactericidal effects at concentrations higher than 20 µL/mL, a finding that was also evident in the case of the BM and BMC films.

### 3.4 Screening Assays of Migration Compounds

**FIGURE 5:**
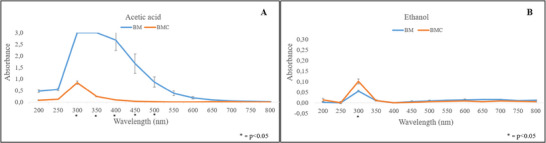
Screening of migration assays in simulating aqueous (3% acetic acid solution) (A) and fatty foods (95% ethanol solution) (B) of BM and BMC films. BM, babassu mesocarp film; BMC, babassu mesocarp film with cake.

The screening revealed distinct migration patterns for BM and BMC films (Figure 5). In 95% (v/v) ethanol, both films released compounds in the UV range (250–400 nm), with BMC showing clearer peaks, which typically correspond to low‐molecular‐weight migrants (Matheus et al. 2024; Zhu et al. 2022). In 3% (v/v) acetic acid, BM showed a more pronounced loss of dark color in the UV region, suggesting increased migration and lower stability in acidic conditions. The stronger migration often observed in acidic simulants has been related to hydrolysis‐driven degradation and partial disintegration of biopolymer matrices, particularly in hydrophilic films obtained by casting, which are prone to swelling and structural relaxation (Franco dos Santos et al. 2023).

Migration behavior depends on polymer and migrant chemistry, matrix morphology (e.g., free volume, crystallinity, cross‐link density), and medium characteristics (e.g., polarity, pH, and fat content), as well as water‐driven plasticization and the coupled kinetics of molecular diffusion and matrix relaxation in the dilated polymeric network (Gupta et al. 2024; Franco dos Santos et al. 2023; Tang et al. 2020). Incorporating BMC served as both a reinforcing and diffusional barrier agent, increasing YM and TS at break while decreasing elongation, likely by restricting segmental mobility and increasing diffusional tortuosity (Tang et al. 2020). In 3% (v/v) acetic acid, BMC exhibited lower overall migration, attributable to reduced sorption/swelling and to intermolecular interactions between BMC constituents and the starch‐rich BM matrix. In ethanol, a slight increase in migration was observed, consistent with extraction of lipophilic fractions present in the BMC and ethanol's higher affinity for less polar microdomains. These findings indicate that BMC simultaneously enhances the mechanical performance and barrier diffusional properties of the film under acidic conditions.

Overall migration tests measure the total amount of substances that can transfer from polymer films into food or food simulants; however, additional analyses are needed to identify the specific migrants in both films. Nevertheless, the present results indicate that BMC maintained structural integrity in contact with both simulants, supporting its potential application in non‐acidic, acidic, fatty, and fatty aqueous foods (Foong et al. 2025; Franco dos Santos et al. 2023).

### 3.5 Thermal Properties and Heat Seal Strength

**FIGURE 6:**
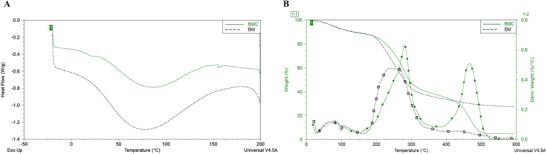
DSC (A) and TGA (B) analysis of BM and BMC films. BM, babassu mesocarp film; BMC, babassu mesocarp film with cake.

The DSC results are described in Figure 6 and Table 4. The incorporation of cake into the BM matrix increased the compositional complexity of the BMC films and introduced additional lignocellulosic components that altered the molecular interactions and thermal behavior. DSC analysis revealed a lower Δ*H* (∼126 J/g) for BMC, indicating a lower energy requirement for phase transition, and a higher *T*_g_ (∼84°C), suggesting greater resistance to thermal softening compared to BM (∼325.5 J/g, ∼76°C). These thermal results are consistent with the TGA data (Figure 6 and Table 5), which according to De Farias et al. (2021) and Rojas‐Lema et al. (2023) show glycerol degradation at around 237–242°C, whereas saccharide degradation begins in the 200–250°C range (Santana and Bonomo 2024), highlighting hemicellulose degradation, which occurs between 200°C and 350°C (El‐Sayed et al. 2024). This is related to the last BM film event at 237°C, where mass loss is highest. Cellulose degradation occurs between 280°C and 400°C (Khotsaeng et al. 2023), and starch degradation occurs between 270°C and 450°C (Santana and Bonomo 2024), which explains the second event at 281°C and the greater mass loss of the BMC film. In addition, the decomposition of lignocellulosic compounds is associated with the third event at 467°C (De Farias et al. 2021; Khotsaeng et al. 2023; El‐Sayed et al. 2024), resulting in a high mass loss. Mechanically, the BMC films exhibited higher stiffness and breaking strength but lower elongation than BM films, reflecting the reinforcing effect of the added lignocellulose fraction. Overall, the integration of BMC improved the thermal resistance and structural robustness of the films, albeit at the expense of flexibility.

**TABLE 4:** Extracted data of babassu mesocarp film with cake (BMC) and babassu mesocarp film (BM) from differential scanning calorimetry (DSC).

Film	*T*_o_ (°C)	*T*_g_ (°C)	∆*H* (J/g)	*T*_o_ (°C)	*T*_g_ (°C)	∆*H* (J/g)	*T*_o_ (°C)	*T*_g_ (°C)	∆*H* (J/g)	
BMC	13.54	21.0	0.767	33.94	84.48	126.1	154.97	155.56		0.639
BM	14.98	—	—	—	76.28	325.5	—	—		—

*Note*: *T*_o_: initial temperature; *T*_g_: glass transition temperature; ∆*H*: transition enthalpy.

**TABLE 5:** Extracted data of babassu mesocarp film with cake (BMC) and babassu mesocarp film (BM) from thermogravimetric analysis (TGA).

Film	*T*1 (°C)	*M*1 (%)	*T*2 (°C)	*M*2 (%)	*T*3 (°C)	*M*3 (%)	*T*4 (°C)	*M*4 (%)	*T*5 (°C)	*M*5 (%)
BMC	71.25	9.53	—	—	281.04	51.62	—	—	467.27	33.13
BM	71.25	10.50	—	—	236.93	51.49	—	—	—	—

*Note*: *T*: temperature at which the event occurred; *M*: percentage of mass lost.

Both films can be sealed, but the sealing strength was found to be weak (BM film = 0.009 ± 0.02 N/mm and BMC film = 0.008 ± 0.02 N/mm), possibly due to the untangling of the polymer molecules caused by the low temperature and time in the sealing process, as the films cannot withstand higher temperatures and heating times as they burn quickly (Das and Chowdhury 2016). The films had a relatively low seal strength, which is generally considered a limitation for conventional food packaging due to the risk of leakage and reduced protection of the product. In certain contexts, however, this property can be reinterpreted as a functional advantage. For single‐use sachets containing products such as sauces, sugar, or oil, the reduced seal strength makes them easier to open, improving convenience and accessibility for the consumer. This property is particularly advantageous for older people, children, and people with limited mobility. Although the films are not universally applicable, they show practical relevance for niche applications where quick and effortless access is desirable. Future studies could explore strategies such as multilayer configurations, surface coatings, or blending with complementary biopolymers to improve sealability and extend the potential of the films to a wider range of food packaging applications.

### 3.6 Packaged Pesto Sauce as a Real System Model, Sensory Analysis, and Puncture Test of the Sachets With Packaged Pesto

**FIGURE 7:**
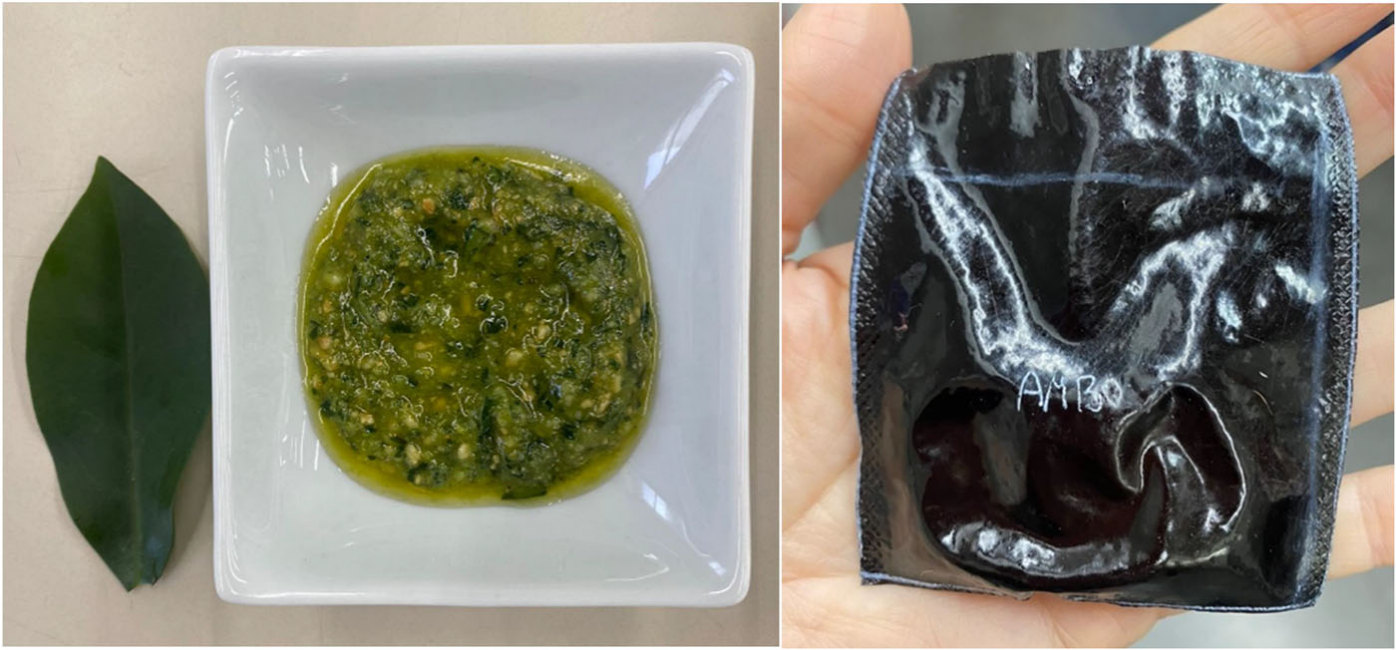
Pesto sauce made with ora‐pro‐nóbis that was packaged in the BMC films.

The physicochemical and microbiological characterization of the pesto (Figure 7) packaged in BMC sachets and stored under different conditions is shown in Table 6. Throughout the experimental period, variations in pH were observed in the range of 5.06–5.48, close to the pH values of commercial pesto (pH 4.00–5.64) (Nicosia et al. 2021).

**TABLE 6:** Characterization of pesto sauce at room temperature (25°C ± 2°C) and refrigerated (4°C ± 2°C) for 4 days.

	pH	L*	a*	b*	∆*E*	Peroxide index (%)	*Salmonella*	Thermotolerant coliforms (NMP/g)	Molds and yeasts (CFU/g)	Thickness (mm)	Puncture force (N)
**Day 0**											
ROOT	5.06 ± 0.0^d^	39.84 ± 0.01^b^	−0.83 ± 0.01^f^	24.95 ± 0.17^b^	Control	0.23 ± 0.02^a^	Absent	<3.0^a^	<10^2a^	0.34 ± 0.02^a^	30.67 ± 5.53^a^
REFT	5.45 ± 0.0^a^	40.94 ± 0.13^a^	−2.29 ± 0.02^d^	26.22 ± 0.20^a^	Control	0.23 ± 0.02^a^	Absent	<3.0^a^	<10^2a^	0.35 ± 0.03^a^	31.07 ± 3.42^a^
**Day 2**											
ROOT	5.48 ± 0.0^a^	24.49 ± 0.02^d^	1.20 ± 0.04^b^	20.71 ± 0.05^d^	16.05 ± 0.16^b^	0.24 ± 0.02^a^	Absent	<3.0^a^	<10^2a^	0.36 ± 0.02^a^	14.86 ± 2.44^b^
REFT	5.45 ± 0.0^a^	25.62 ± 0.07^c^	−1.92 ± 0.07^e^	16.04 ± 0.13^f^	18.40 ± 0.21^a^	0.17 ± 0.02^a,c^	Absent	<3.0^a^	<10^2a^	0.34 ± 0.02^a^	21.77 ± 4.10^b^
**Day 4**											
ROOT	5.30 ± 0.0^b^	25.74 ± 0.04^c^	2.21 ± 0.06^a^	23.50 ± 0.14^c^	14.49 ± 0.17^c^	0.27 ± 0.02^a,b^	Absent	<3.0^a^	<10^2a^	0.37 ± 0.02^a^	38.77 ± 7.83^a^
REFT	5.22 ± 0.0^c^	25.39 ± 0.70^c,d^	0.73 ± 0.04^c^	19.73 ± 0.01^e^	17.24 ± 0.98^a,b^	0.22 ± 0.01^a^	Absent	<3.0^a^	<10^2a^	0.36 ± 0.03^a^	28.08 ± 4.85^a^

*Note*: ROOT: room temperature (25°C ± 2°C) and REFT: refrigerated temperature (4°C ± 2°C). Values with different letters are significantly different according to Tukey's test (*p* < 0.05).

Regarding the L* value, the unrefrigerated pesto was darker on the first and second day, possibly due to the increased storage temperature and chlorophyll degradation (Glicerina et al. 2023). On the fourth day, the pesto stored at room temperature showed a delayed darkening process, whereas the refrigerated pesto showed the same luminosity. These two samples showed similar brightness values, which were comparable to those of the refrigerated pesto on the second day.

The a* value showed a reduction depending on the type of storage, with the refrigerated pesto consistently showing greener. From the second day onward, the green coloration of the sauce stored at room temperature decreased noticeably and also reached a pronounced level in the refrigerated sample on the fourth day. This observation is consistent with the results of Zardetto and Barbanti (2020). The b* value indicates that the yellowish pigments from the sauce ingredients predominate. Finally, the discrepancy in color was less pronounced in the pesto stored at room temperature (25°C ± 2°C).

Throughout the analysis, peroxide levels remained within the parameters established by Brazilian legislation (Brasil, Ministério da Agricultura, Pecuária e Abastecimento 2012) and showed no statistically significant difference from the values observed on Day 0, regardless of the type of storage. Likewise, microbiological analysis showed that the pesto sauces were free of *Salmonella* and not positive for thermotolerant coliforms (<3.0 MPN/g), indicating good hygiene and manufacturing practices (Hammad et al. 2022). In addition, the differences in storage had no effect on the growth of molds and yeasts, which were found at all check points <10^2^ CFU/g.

As for the sachets during storage, no significant differences in thickness were observed on the different days and types of storage. The same result was observed in the puncture test, where the maximum puncture force remained constant, except for Day 2, when the sachets required less force than the others. This discrepancy in the force required is not due to the storage method or checkpoint, but rather the inherent variability of the films produced by the casting process, as previously observed by Paiva et al. (2024). Overall, the sachets showed excellent puncture resistance at 25°C ± 2°C (∼28.10 N) and under refrigeration, 4°C ± 2°C (∼26.97 N). This result contrasts with previous studies reporting lower puncture forces, such as films of Cipó‐titica and andiroba oil (6.70 N; Paiva et al. 2024), corn starch in combination with chitosan (10.81 N; Bof et al. 2021) and red prickly pear bark powder and its extract (17.7 N; Aparicio‐Fernández et al. 2018). The short test period for the sachets complies with the Brazilian Health Regulatory Agency (ANVISA) (RDC 216/2004; “Guide for Determining Food Shelf Life”), and the FDA Food Code (2022). This is therefore an initial assessment. To achieve a longer shelf life for commercial purposes, further reformulations of the sauce (e.g., with natural additives, processing technologies, or other packaging strategies) and additional shelf‐life studies would be required.

**FIGURE 8:**
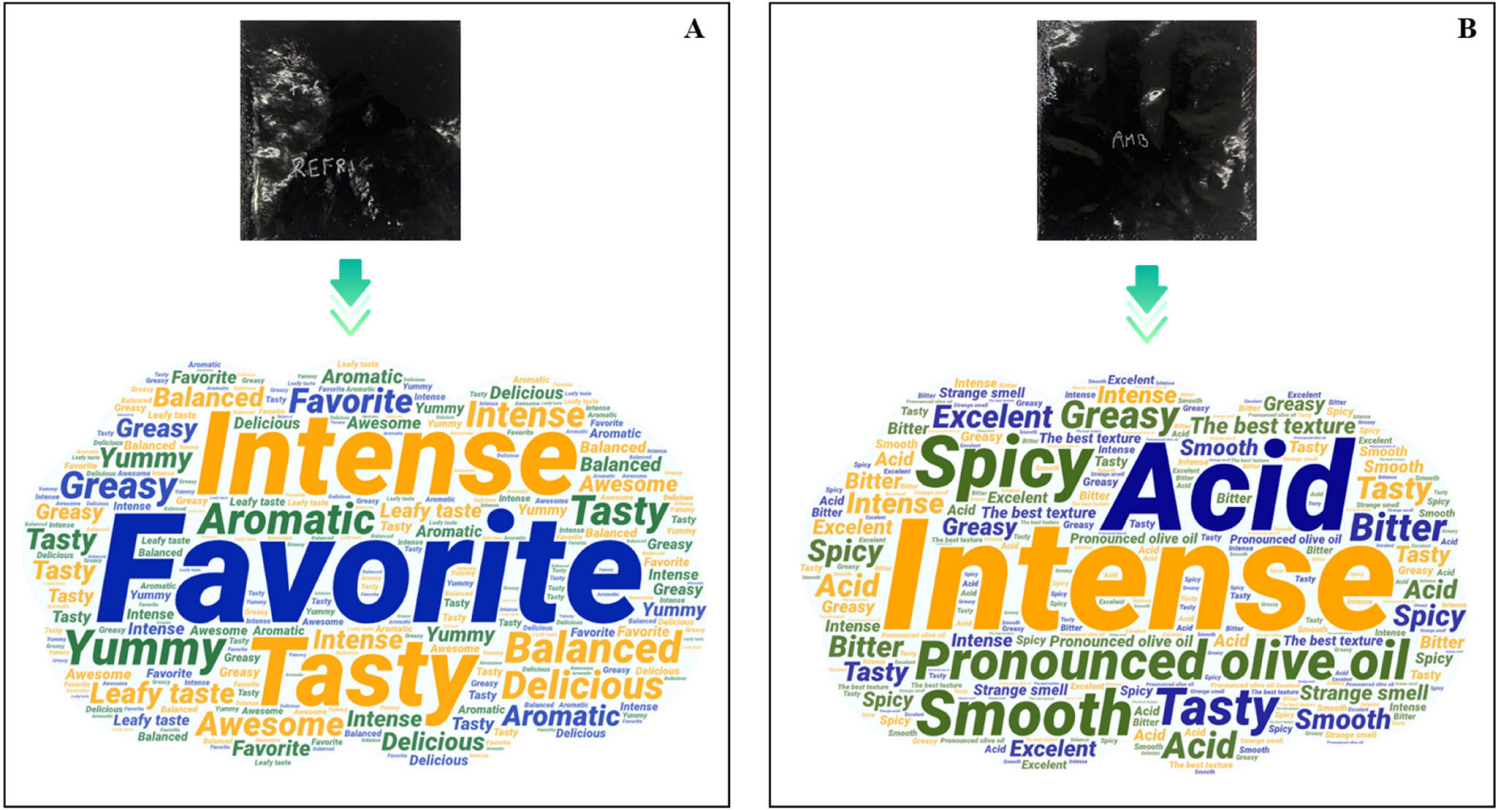
Word cloud of tasters’ comments on refrigerated pesto (A) (4°C ± 2°C) and pesto at room temperature (B) (25°C ± 2°C).

The free comments on the impressions of the pestos are shown in Figure 8. The attributes evaluated were appearance, aroma, texture, taste and overall acceptability as well as purchase intention. Panelists were between 18 and 69 years old, and 35% stated that they had never eaten pesto before.

According to the open comments of the panelists, flavor was the attribute that showed the greatest difference between the two pesto samples when stored at 25°C and 4°C. However, on the acceptability scale, aroma was the deciding factor between the two sauces. The refrigerated pesto showed higher acceptability, with all attributes scoring 8 (I liked it very much) on the acceptability scale and 4 (I would probably buy it) in terms of purchase intention. The room temperature pesto, on the other hand, received the same scores for acceptability and purchase intention as the other sauce, except for aroma, which was rated 7 (I liked it moderately). Although most respondents were not familiar with this type of sauce, there was generally a high level of acceptability for both types of storage.

## 4 Conclusion

The present study shows the potential of BM in combination with the supernatant of BMC as promising raw materials for the development of bio‐based films. Incorporation of the BMC into the mesocarp matrix significantly improved TS and stiffness, with BMC films exhibiting higher TS and YM than BM films, but lower flexibility, reflecting the classical trade‐off between stiffness and elongation in composite biomaterials. Both BM and BMC films exhibited a distinct dark coloration, likely due to residual lignin and hemicellulose as well as Maillard and caramelization reactions during alkaline and thermal treatment. Apart from visual appeal, this feature reduced light transmission and gave the BMC films excellent UV blocking ability, providing better protection in the visible spectrum. The films maintained their pigment stability both under refrigeration and at room temperature (25°C). This contrasts with the fading that normally occurs with pigment‐based films and highlights their potential for consistent performance. The films showed a strong biodisintegration capacity, with the rate of degradation dependent on formulation and environment. In soil, higher microbial activity, moisture and temperature accelerated the degradation of the carbohydrate‐rich BM films, whereas protein‐ and polymer‐enriched BMC films degraded more slowly but released more nutrients. In aquatic systems, seawater promoted faster BMC degradation probably through ionic interactions and structural heterogeneity, whereas freshwater showed no formulation‐dependent differences. Importantly, no phytotoxic effects were observed, confirming compatibility with soil ecosystems and supporting nutrient cycling in line with circular bioeconomy principles. Both films showed antimicrobial activity against foodborne pathogens, with the BMC films exhibiting a broader spectrum of inhibition. The practical advantages of the BMC films were confirmed by their conversion into sachets for packaging pesto, which remained physicochemically stable and microbiologically safe both when stored at 4°C and at 25°C. The sachets themselves also retained high puncture resistance throughout storage. The sensory evaluation revealed general acceptance, with the refrigerated samples being preferred in terms of flavor retention. Purchase intent was also positive, suggesting that consumers are receptive to the product despite their limited familiarity with pesto. Overall, BM and cake films prove to be multifunctional biocompostable materials that are well suited for single‐use sachets designed to protect light‐sensitive foods.

## Author Contributions

**Letícia de Oliveira Gonçalves**: conceptualization, data curation, formal analysis, investigation, writing – original draft, methodology. **Patrícia Marques De Farias**: conceptualization, data curation, formal analysis, investigation, methodology. **Thalita Ferreira de Freitas**: formal analysis, investigation. **Lilia Zago**: investigation, formal analysis, methodology. **Ricardo Felipe Alves Moreira**: investigation, formal analysis, methodology. **Bianca Chieregato Maniglia**: investigation, writing – review and editing, formal analysis, data curation, resources. **Ana Elizabeth Cavalcante Fai**: formal analysis, resources, writing – review and editing, conceptualization, funding acquisition, project administration, data curation, supervision.

## Funding

The authors are grateful to UNIRIO, UERJ, USP, Coordenação de Aperfeiçoamento de Pessoal de Nível Superior (CAPES) (code 001), and Fundação de Amparo à Pesquisa do Estado do Rio de Janeiro (FAPERJ) for the financial support (code E‐26/201.428/2022; code E‐26/210.326/2022).

## Conflicts of Interest

The authors declare no conflicts of interest.

## References

[jfds70690-bib-0001] Abdillah, A. A. , and A. L. Charles . 2021. “Characterization of a Natural Biodegradable Edible Film Obtained From Arrowroot Starch and Iota‐Carrageenan and Application in Food Packaging.” International Journal of Biological Macromolecules 191, no. August: 618–626. 10.1016/j.ijbiomac.2021.09.141. 34582908

[jfds70690-bib-0002] Abrokwah, S. , B. Ekumah , R. Adade , and I. S. G. Akuoko . 2022. “Drivers of Single‐Use Plastic Waste Generation: Lessons From Packaged Water Consumers in Ghana.” GeoJournal 87, no. 4: 2611–2623. 10.1007/s10708-021-10390-w.

[jfds70690-bib-0003] Akkuş, M. , and M. Budakçı . 2020. “Determination of Color‐Changing Effects of Bleaching Chemicals on Some Heat‐Treated Woods.” Journal of Wood Science 66, no. 1: 68. 10.1186/s10086-020-01916-w.

[jfds70690-bib-0004] Conceição, N. , B. R. Albuquerque , C. Pereira , et al. 2020. “By‐Products of Camu‐Camu [Myrciaria Dubia (Kunth) McVaugh] as Promising Sources of Bioactive High Added‐Value Food Ingredients: Functionalization of Yogurts.” Molecules (Basel, Switzerland) 25: 70. 10.3390/molecules25010070 PMC6982765 31878221

[jfds70690-bib-0005] Aldas, M. , C. Pavon , J. M. Ferri , M. P. Arrieta , and J. López‐Martínez . 2021. “Films Based on Mater‐Bi® Compatibilized With Pine Resin Derivatives: Optical, Barrier, and Disintegration Properties.” Polymers 13, no. 9: 1506. 10.3390/polym13091506. 34067087 PMC8124954

[jfds70690-bib-0006] Alves, Z. , N. M. Ferreira , P. Ferreira , and C. Nunes . 2022. “Design of Heat Sealable Starch‐Chitosan Bioplastics Reinforced With Reduced Graphene Oxide for Active Food Packaging.” Carbohydrate Polymers 291, no. April: 119517. 10.1016/j.carbpol.2022.119517. 35698362

[jfds70690-bib-0007] Aparicio‐Fernández, X. , A. Vega‐Ahuatzin , C. E. Ochoa‐Velasco , S. Cid‐Pérez , P. Hernández‐Carranza , and R. Ávila‐Sosa . 2018. “Physical and Antioxidant Characterization of Edible Films Added With Red Prickly Pear (*Opuntia ficus‐indica* L.) Cv. San Martín Peel and/or Its Aqueous Extracts.” Food and Bioprocess Technology 11, no. 2: 368–379. 10.1007/s11947-017-2017-x.

[jfds70690-bib-0008] ASTM . 2012. Standard Test Method for Tensile Properties of Thin Plastic Sheeting (ASTM Standard D882‐12). American Society for Testing and Materials.

[jfds70690-bib-0009] Barroqueiro, E. S. B. , D. S. Prado , P. S. Barcellos , et al. 2016. “Immunomodulatory and Antimicrobial Activity of Babassu Mesocarp Improves the Survival in Lethal Sepsis.” Evidence‐Based Complementary and Alternative Medicine 2016: 2859652. 10.1155/2016/2859652. 27630733 PMC5007311

[jfds70690-bib-0010] Bof, M. J. , D. E. Locaso , and M. A. García . 2021. “Corn Starch‐Chitosan Proportion Affects Biodegradable Film Performance for Food Packaging Purposes.” Starch/Staerke 73, no. 5–6: 1–12. 10.1002/star.202000104.

[jfds70690-bib-0011] Brasil, Ministério da Agricultura, Pecuária e Abastecimento . 2012. Instrução Normativa no 1/2012 .

[jfds70690-bib-0012] Brasil, Agência Nacional de Vigilância Sanitária (ANVISA) . 2022. Instrução Normativa—IN no 161, de 1o de julho de 2022 .

[jfds70690-bib-0013] De Bruno, A. , A. Gattuso , R. Romeo , S. Santacaterina , and A. Piscopo . 2022. “Functional and Sustainable Application of Natural Antioxidant Extract Recovered From Olive Mill Wastewater on Shelf‐Life Extension of ‘Basil Pesto’.” Applied Sciences 12, no. 21: 10965. 10.3390/app122110965.

[jfds70690-bib-0014] Bugatti, C. , K. Cristine de Almeida , M. S. A. Guimarães , and N. F. G. Amâncio . 2023. “Microplásticos e Nanoplásticos e Sua Relevância Na Saúde Humana: Uma Revisão de Literatura.” Research, Society and Development 12, no. 1: e6712139302. 10.33448/rsd-v12i1.39302.

[jfds70690-bib-0015] Das, M. , and T. Chowdhury . 2016. “Heat Sealing Property of Starch Based Self‐Supporting Edible Films.” Food Packaging and Shelf Life 9: 64–68. 10.1016/j.fpsl.2016.05.002.

[jfds70690-bib-0016] De Farias, P. M. , L. B. Vasconcelos , M. E. S. Ferreira , E. G. A. Filho , V. A. A. De Freitas , and D. R. Tapia‐Blácido . 2021. “Nopal Cladode as a Novel Reinforcing and Antioxidant Agent for Starch‐Based Films: A Comparison With Lignin and Propolis Extract.” International Journal of Biological Macromolecules 183: 614–626. 10.1016/j.ijbiomac.2021.04.143. 33933543

[jfds70690-bib-0017] De Farias, P. M. , L. B. De Vasconcelos , M. E. S. Ferreira , E. G. A. Filho , and D. R. Tapia‐Blácido . 2023. “Use of Chemically Treated Nopal Cladodes as Additive in the Cassava Starch Composite Films.” Journal of Vinyl and Additive Technology 29, no. 6: 1109–1124. 10.1002/vnl.22040.

[jfds70690-bib-0018] Demircan, B. , and Y. S. Velioglu . 2025. “Revolutionizing Single‐Use Food Packaging: A Comprehensive Review of Heat‐Sealable, Water‐Soluble, and Edible Pouches, Sachets, Bags, or Packets.” Critical Reviews in Food Science and Nutrition 65, no. 8: 1497–1517. 10.1080/10408398.2023.2295433. 38117069

[jfds70690-bib-0019] Díaz‐Díaz, E. D. , M. L. M. Haro , A. Patriarca , et al. 2023. “Assessment of the Enhancement Potential of Salicylic Acid on Physicochemical, Mechanical, Barrier, and Biodegradability Features of Potato Starch Films.” Food Packaging and Shelf Life 38, no. June: 1–9. 10.1016/j.fpsl.2023.101108.

[jfds70690-bib-0020] Diaz‐Morales, N. , M. Ortega‐Heras , A. M. Diez‐Maté , M. L. Gonzalez‐SanJose , and P. Muñiz . 2022. “Antimicrobial Properties and Volatile Profile of Bread and Biscuits Melanoidins.” Food Chemistry 373, no. pt. B: 131648. 10.1016/j.foodchem.2021.131648. 34839966

[jfds70690-bib-0021] Dirpan, A. , A. Fadiah Ainani , and M. Djalal . 2023. “A Review on Biopolymer‐Based Biodegradable Film for Food Packaging: Trends Over the Last Decade and Future Research.” Polymers 15, no. 13: 2781. 10.3390/polym15132781. 37447428 PMC10346960

[jfds70690-bib-0022] El‐Sayed, S. A. , T. M. Khass , and M. E. Mostafa . 2024. “Thermal Degradation Behaviour and Chemical Kinetic Characteristics of Biomass Pyrolysis Using TG/DTG/DTA Techniques.” Biomass Conversion and Biorefinery 14, no. 15: 17779–17803. 10.1007/s13399-023-03926-2.

[jfds70690-bib-0023] Ferrari, R. A. , and M. P. Soler . 2015. “Obtention and Characterization of Coconut Babassu Derivatives.” Scientia Agricola 72, no. 4: 291–296. 10.1590/0103-9016-2014-0278.

[jfds70690-bib-0024] Filipini, G. S. , V. P. Romani , and V. G. Martins . 2020. “Biodegradable and Active‐Intelligent Films Based on Methylcellulose and Jambolão (*Syzygium cumini*) Skins Extract for Food Packaging.” Food Hydrocolloids 109, no. May: 106139. 10.1016/j.foodhyd.2020.106139.

[jfds70690-bib-0025] Foong, H. L. , R. Sulaiman , E. M. Azman , et al. 2025. “Development and Characterisation of Polylactic Acid/Cinnamon Bark Oil Films: Phenolic Migration Into Various Food Simulants.” Food Packaging and Shelf Life 48: 101455. 10.1016/j.fpsl.2025.101455.

[jfds70690-bib-0026] Franco dos Santos, L. , B. Biduski , S. T. Lopes , T. E. Bertolin , and L. R. Santos . 2023. “Brazilian Native Fruit Pomace as a Source of Bioactive Compounds on Starch‐Based Films: Antimicrobial Activities and Food Simulator Release.” International Journal of Biological Macromolecules 242, no. pt. 2: 124900. 10.1016/j.ijbiomac.2023.124900. 37201884

[jfds70690-bib-0027] Garcia, J. A. A. , R. C. G. Corrêa , L. Barros , et al. 2019. “Chemical Composition and Biological Activities of Juçara (*Euterpe edulis Martius*) Fruit By‐Products, a Promising Underexploited Source of High‐Added Value Compounds.” Journal of Functional Foods 55, no. February: 325–332. 10.1016/j.jff.2019.02.037.

[jfds70690-bib-0028] Gasparini, S. P. , F. B. Ribeiro , J. C. Siqueira , M. A. D. Bomfim , and D. C. N. Nascimento . 2015. “Avaliação Nutricional Da Torta de Babaçu Para Frangos de Crescimento Lento Em Diferentes Idades.” Revista Caatinga 28, no. 2: 126–134.

[jfds70690-bib-0029] Glicerina, V. , L. Siroli , D. Gottardi , et al. 2023. “Influence of an Innovative, Biodegradable Active Packaging on the Quality of Sunflower Oil and ‘Pesto’ Sauce During Storage.” Applied Food Research 3, no. 2: 100313. 10.1016/j.afres.2023.100313.

[jfds70690-bib-0030] Gupta, R. K. , S. Pipliya , S. Karunanithi , et al. 2024. “Migration of Chemical Compounds From Packaging Materials Into Packaged Foods: Interaction, Mechanism, Assessment, and Regulations.” Foods 13: 3125. 10.3390/foods13193125. 39410160 PMC11475518

[jfds70690-bib-0031] Guo, G. , A. Xiang , and H. Tian . 2018. “Thermal and Mechanical Properties of Eco‐Friendly Poly(Vinyl Alcohol) Films With Surface Treated Bagasse Fibers.” Journal of Polymers and the Environment 26, no. 9: 3949–3956. 10.1007/s10924-018-1270-z.

[jfds70690-bib-0032] Hammad, A. M. , A. Eltahan , H. A. Hassan , N. H. Abbas , H. Hussien , and T. Shimamoto . 2022. “Loads of Coliforms and Fecal Coliforms and Characterization of Thermotolerant *Escherichia coli* in Fresh Raw Milk Cheese.” Foods 11, no. 3: 332. 10.3390/foods11030332. 35159482 PMC8834472

[jfds70690-bib-0033] Instituto Adolf Lutz . 2008. Métodos Físico‐Químicos para Análise de Alimentos. 4th ed. Instituto Adolf Lutz.

[jfds70690-bib-0034] Jagadeesh, P. , M. Puttegowda , S. M. Rangappa , and S. Siengchin . 2021. “A Review on Extraction, Chemical Treatment, Characterization of Natural Fibers and Its Composites for Potential Applications.” Polymer Composites 42, no. 12: 6239–6264. 10.1002/pc.26312.

[jfds70690-bib-0035] Khotsaeng, N. , W. Simchuer , T. Imsombut , and P. Srihanam . 2023. “Effect of Glycerol Concentrations on the Characteristics of Cellulose Films From Cattail (*Typha angustifolia* L.) Flowers.” Polymers 15, no. 23: 1–13. 10.3390/polym15234535. PMC10708089 38231905

[jfds70690-bib-0036] Li, W. , Y. Zhao , W. Sun , T. Dong , M. D. A. Saldaña , and W. Sun . 2022. “Multi‐Responsive Poly N‐Isopropylacrylamide/Poly N‐Tert‐Butylacrylamide Nanocomposite Hydrogel With the Ability to Be Adsorbed on the Chitosan Film as an Active Antibacterial Material.” International Journal of Biological Macromolecules 208, no. April: 1019–1028. 10.1016/j.ijbiomac.2022.03.198. 35381289

[jfds70690-bib-0037] Lima, M. A. , L. D. Gomez , C. G. Steele‐King , et al. 2014. “Evaluating the Composition and Processing Potential of Novel Sources of Brazilian Biomass for Sustainable Biorenewables Production.” Biotechnology for Biofuels 7, no. 1: 10. 10.1186/1754-6834-7-10. 24438499 PMC4028816

[jfds70690-bib-0038] Lira, M. M. , J. Gonçalves de Oliveira Filho , T. Leal de Sousa , et al. 2023. “Selected Plants Producing Mucilage: Overview, Composition, and Their Potential as Functional Ingredients in the Development of Plant‐Based Foods.” Food Research International 169, no. April: 112822. 10.1016/j.foodres.2023.112822. 37254398

[jfds70690-bib-0039] Lobiuc, A. , N. E. Pavăl , I. I. Mangalagiu , et al. 2023. “Future Antimicrobials: Natural and Functionalized Phenolics.” Molecules (Basel, Switzerland) 28, no. 3: 1114. 10.3390/molecules28031114. 36770780 PMC9920704

[jfds70690-bib-0040] Silva, A. G. M. , I. Borges , J. N. Neiva , et al. 2008. Degradabilidade in Situ Da Torta De Babaçu‐Frações Fibrosas. EMBRAPA.

[jfds70690-bib-0042] Maniglia, B. C. , L. Tessaro , A. A. Lucas , and D. R. Tapia‐Blácido . 2017. “Bioactive Films Based on Babassu Mesocarp Flour and Starch.” Food Hydrocolloids 70: 383–391. 10.1016/j.foodhyd.2017.04.022.

[jfds70690-bib-0043] Matheus, J. R. V. , C. Maragoni‐Santos , T. Ferreira de Freitas , et al. 2024. “Starch‐Pectin Smart Tag Containing Purple Carrot Peel Anthocyanins as a Potential Indicator of Analogous Meat Freshness.” International Journal of Biological Macromolecules 283, no. pt. 1: 137161. 10.1016/j.ijbiomac.2024.137161. 39500436

[jfds70690-bib-0044] Matheus, J. R. V. , T. B. B. Nogueira , A. P. A. Pereira , et al. 2021. “Antibacterial Films Made With Persimmon (*Diospyros kaki* L.), Pectin, and Glycerol: An Experimental Design Approach.” Journal of Food Science 86, no. 10: 4539–4553. 10.1111/1750-3841.15886. 34431096

[jfds70690-bib-0045] Medina‐Jaramillo, C. , O. Ochoa‐Yepes , C. Bernal , and L. Famá . 2017. “Active and Smart Biodegradable Packaging Based on Starch and Natural Extracts.” Carbohydrate Polymers 176, no. May: 187–194. 10.1016/j.carbpol.2017.08.079. 28927597

[jfds70690-bib-0046] Mirres, A. C. M. , I. R. S. Vieira , L. Tessaro , et al. 2024. “Nanocomposite Films of Babassu Coconut Mesocarp and Food Packaging.” Foods 13: 1–16. 10.3390/foods13121895 PMC11203357 38928835

[jfds70690-bib-0047] Mohammadalinejhad, S. , H. Almasi , and M. Moradi . 2020. “Immobilization of Echium Amoenum Anthocyanins Into Bacterial Cellulose Film: A Novel Colorimetric PH Indicator for Freshness/Spoilage Monitoring of Shrimp.” Food Control 113, no. February: 107169. 10.1016/j.foodcont.2020.107169.

[jfds70690-bib-0048] Momen, S. , F. Alavi , and M. Aider . 2021. “Alkali‐Mediated Treatments for Extraction and Functional Modification of Proteins: Critical and Application Review.” Trends in Food Science and Technology 110, no. October: 778–797. 10.1016/j.tifs.2021.02.052.

[jfds70690-bib-0049] Nicosia, C. , P. Fava , A. Pulvirenti , A. Antonelli , and F. Licciardello . 2021. “Domestic Use Simulation and Secondary Shelf Life Assessment of Industrial *Pesto alla genovese* .” Foods 10, no. 8: 1948. 10.3390/foods10081948. 34441725 PMC8391206

[jfds70690-bib-0050] Nogueira, D. , and V. G. Martins . 2019. “Use of Different Proteins to Produce Biodegradable Films and Blends.” Journal of Polymers and the Environment 27, no. 9: 2027–2039. 10.1007/s10924-019-01494-z.

[jfds70690-bib-0051] Nouraddini, M. , M. Esmaiili , and F. Mohtarami . 2018. “Development and Characterization of Edible Films Based on Eggplant Flour and Corn Starch.” International Journal of Biological Macromolecules 120: 1639–1645. 10.1016/j.ijbiomac.2018.09.126. 30248421

[jfds70690-bib-0052] Paiva, C. S. , F. G. Batista , D. W. Silva , et al. 2024. “Andiroba Oil (*Carapa guianensis* Aublet) as a Functionalizing Agent for Titica Vine (*Heteropsis flexuosa*) Nanofibril Films: Biodegradable Products From Species Native to the Amazon Region.” Sustainability 16, no. 11: 4395. 10.3390/su16114395.

[jfds70690-bib-0053] Pasquet, P. L. , D. Julien‐David , M. Zhao , M. Villain‐Gambier , and D. Trébouet . 2024. “Stability and Preservation of Phenolic Compounds and Related Antioxidant Capacity From Agro‐Food Matrix: Effect of PH and Atmosphere.” Food Bioscience 57, no. January: 103586. 10.1016/j.fbio.2024.103586.

[jfds70690-bib-0054] Pereira, D. G. M. , J. M. Vieira , A. A. Vicente , and R. M. S. Cruz . 2021. “Development and Characterization of Pectin Films With *Salicornia ramosissima*: Biodegradation in Soil and Seawater.” Polymers 13, no. 16: 2632. 10.3390/polym13162632. 34451172 PMC8398948

[jfds70690-bib-0055] Petkoska, A. T. , D. Daniloski , N. M. D'Cunha , N. Naumovski , and A. T. Broach . 2021. “Edible Packaging: Sustainable Solutions and Novel Trends in Food Packaging.” Food Research International 140, no. May: 109981. 10.1016/j.foodres.2020.109981. 33648216

[jfds70690-bib-0056] Rangel‐Buitrago, N. , F. Galgani , and W. J. Neal . 2024. “Navigating Between Socio‐Economic Viability and Environmental Impacts: The Sachets and Sticks Paradox.” Science of the Total Environment 920, no. February: 171022. 10.1016/j.scitotenv.2024.171022. 38367726

[jfds70690-bib-0057] Raposo, A. K. S. , L. C. Paixão , A. A. Rocha , et al. 2021. “Characterization of Biodegradable Films Produced From Mixtures of Alginate, Starch and Babassu Fibers.” Journal of Polymers and the Environment 29, no. 4: 1212–1226. 10.1007/s10924-020-01952-z.

[jfds70690-bib-0058] Rêgo, M. T. C. , J. R. A. Barros , F. Angelotti , N. D. Costa , and B. F. Dantas . 2017. Germinação De Sementes De Cebola Em Diferentes Concentrações de Co2 E Temperatura. Portal EMBRAPA.

[jfds70690-bib-0059] Reichert, A. A. , T. C. D. E. Freitas , J. H. Alano , and A. D. De Oliveira . 2022. “Evaluation of Phytotoxicity and Biodegradation of Cellulose Reinforced Starch Biocomposites.” Cellulose Chemistry and Technology 56, no. 7–8: 807–814. 10.35812/CelluloseChemTechnol.2022.56.72.

[jfds70690-bib-0060] Rodrigues, S. C. S. , A. S. Silva , L. H. Carvalho , T. S. Alves , and R. Barbosa . 2020. “Morphological, Structural, Thermal Properties of a Native Starch Obtained From Babassu Mesocarp for Food Packaging Application.” Journal of Materials Research and Technology 9, no. 6: 15670–15678. 10.1016/j.jmrt.2020.11.030.

[jfds70690-bib-0061] Rojas‐Lema, S. , K. Nilsson , M. Langton , et al. 2023. “The Effect of Pine Cone Lignin on Mechanical, Thermal and Barrier Properties of Faba Bean Protein Films for Packaging Applications.” Journal of Food Engineering 339, no. July: 111282. 10.1016/j.jfoodeng.2022.111282.

[jfds70690-bib-0062] Samir, A. , F. H. Ashour , A. A. Abdel Hakim , and M. Bassyouni . 2022. “Recent Advances in Biodegradable Polymers for Sustainable Applications.” Nature 6, no. 1: 68. 10.1038/s41529-022-00277-7.

[jfds70690-bib-0063] Santana, R. F. , and R. C. F. Bonomo . 2024. “Thermal Analysis for Evaluation of Biodegradable Films: A Review.” Journal of Thermal Analysis and Calorimetry 149, no. 14: 7155–7168. 10.1007/s10973-024-13339-6.

[jfds70690-bib-0064] Santos, L. B. , R. D. Silva , J. D. Alonso , et al. 2023. “Bioplastics From Orange Processing Byproducts by an Ecoefficient Hydrothermal Approach.” Food Packaging and Shelf Life 38, no. May: 101114. 10.1016/j.fpsl.2023.101114.

[jfds70690-bib-0065] Santos‐Filho, L. G. A. , K. N. C. Castro , A. M. L. Pereira , and F. M. Diniz . 2019. Detecção Da Atividade Antibacteriana In Vitro de Compostos Naturais à Base de Plantas: Metodologia Científica. Teresina: Embrapa Meio‐Norte. Teresina.

[jfds70690-bib-0066] Sanyang, M. L. , S. M. Sapuan , M. Jawaid , M. R. Ishak , and J. Sahari . 2015. “Effect of Plasticizer Type and Concentration on Tensile, Thermal and Barrier Properties of Biodegradable Films Based on Sugar Palm (*Arenga pinnata*) Starch.” Polymers 7, no. 6: 1106–1124. 10.3390/polym7061106. PMC4711441 26787952

[jfds70690-bib-0068] Silva, L. S. , B. D. Ribeiro , and I. Itabaiana . 2023. “Investigation of Babassu Mesocarp Dissolution in the Presence of Deep Eutectic Solvents.” Bioenergy Research 16, no. 4: 2081–2092. 10.1007/s12155-023-10692-6.

[jfds70690-bib-0069] Silva, R. D. , T. F. Pacheco , A. D. De Santi , et al. 2024. “From Bulk Banana Peels to Active Materials: Slipping Into Bioplastic Films With High UV‐Blocking and Antioxidant Properties.” Journal of Cleaner Production 438: 140709. 10.1016/j.jclepro.2024.140709.

[jfds70690-bib-0070] Silva, S. H. , I. C. O. Neves , N. L. Oliveira , et al. 2019. “Extraction Processes and Characterization of the Mucilage Obtained From Green Fruits of *Pereskia aculeata* Miller.” Industrial Crops and Products 140, no. August: 111716. 10.1016/j.indcrop.2019.111716.

[jfds70690-bib-0071] Sioriki, E. , V. Lemarcq , F. Alhakim , et al. 2021. “Impact of Alkalization Conditions on the Phytochemical Content of Cocoa Powder and the Aroma of Cocoa Drinks.” LWT—Food Science and Technology 145, no. March: 111181. 10.1016/j.lwt.2021.111181.

[jfds70690-bib-0072] Song, G. , X. Li , and R. Hui . 2017. “Effect of Biological Soil Crusts on Seed Germination and Growth of an Exotic and Two Native Plant Species in an Arid Ecosystem.” PLoS ONE 12, no. 10: 1–16. 10.1371/journal.pone.0185839. PMC5627943 28977018

[jfds70690-bib-0073] Tang, Z. , F. Fan , C. Fan , K. Jiang , and Y. Qin . 2020. “The Performance Changes and Migration Behavior of PLA/Nano‐TiO_2_ Composite Film by High‐Pressure Treatment in Ethanol Solution.” Polymers 12, no. 2: 471. 10.3390/polym12020471. 32085498 PMC7077698

[jfds70690-bib-0074] Tan, J. , T. Liu , Y. Yao , et al. 2021. “Changes in Physicochemical and Antioxidant Properties of Egg White During the Maillard Reaction Induced by Alkali.” LWT—Food Science and Technology 143, no. February: 111151. 10.1016/j.lwt.2021.111151.

[jfds70690-bib-0075] Tripathi, D. K. , V. P. Singh , D. K. Chauhan , S. M. Prasad , and N. K. Dubey . 2014. “Role of Macronutrients in Plant Growth and Acclimation: Recent Advances and Future Prospective.” In Improvement of Crops in the Era of Climatic Changes, edited by P. Ahmad , M. Wani , M. Azooz , L. S. Phan Tran , 1–14. Springer.

[jfds70690-bib-0076] Velasquez, S. T. R. , Q. Hu , J. Kramm , V. C. Santin , C. Völker , and F. R. Wurm . 2025. “Plastics of the Future? An Interdisciplinary Review on Biobased and Biodegradable Polymers: Progress in Chemistry, Societal Views, and Environmental Implications.” Angewandte Chemie—International Edition 64, no. 23: e202423406. 10.1002/anie.202423406. 40126932

[jfds70690-bib-0077] Weisbrich, M. , D. Messerer , F. Holzer , U. Trommler , U. Roland , and K. Holschemacher . 2024. “The Impact of Liquids and Saturated Salt Solutions on Polymer‐Coated Fiber Optic Sensors for Distributed Strain and Temperature Measurement.” Sensors 24, no. 14: 4659. 10.3390/s24144659. 39066056 PMC11281284

[jfds70690-bib-0079] Worku, L. A. , R. K. Bachheti , A. Bachheti , T. S. Milessi , and A. K. Chandel . 2024. Understanding the Biochemical Changes at Molecular Level During Biomass Pretreatment: A Comprehensive Analysis. Cellulose. Vol. 31. Springer Netherlands. 10.1007/s10570-024-06081-7.

[jfds70690-bib-0080] Ying, W. , Z. Shi , H. Yang , G. Xu , Z. Zheng , and J. Yang . 2018. “Effect of Alkaline Lignin Modification on Cellulase‐Lignin Interactions and Enzymatic Saccharification Yield.” Biotechnology for Biofuels 11, no. 1: 1–13. 10.1186/s13068-018-1217-6. 30083227 PMC6069831

[jfds70690-bib-0081] Zardetto, S. , and D. Barbanti . 2020. “Shelf Life Assessment of Fresh Green Pesto Using an Accelerated Test Approach.” Food Packaging and Shelf Life 25: 100524. 10.1016/j.fpsl.2020.100524.

[jfds70690-bib-0082] Zborowska, M. , H. Waliszewska , B. Waliszewska , S. Borysiak , J. Brozdowski , and A. Stachowiak‐Wencek . 2022. “Conversion of Carbohydrates in Lignocellulosic Biomass After Chemical Pretreatment.” Energies 15, no. 1: 1–14. 10.3390/en15010254.

[jfds70690-bib-0083] Zhang, Y. , and W. Jiang . 2023. “Effective Strategies to Enhance Ultraviolet Barrier Ability in Biodegradable Polymer‐Based Films/Coatings for Fruit and Vegetable Packaging.” Trends in Food Science and Technology 139, no. August: 104139. 10.1016/j.tifs.2023.104139.

[jfds70690-bib-0084] Zhang, J. , X. Zou , X. Zhai , X. W. Huang , C. Jiang , and M. Holmes . 2019. “Preparation of an Intelligent PH Film Based on Biodegradable Polymers and Roselle Anthocyanins for Monitoring Pork Freshness.” Food Chemistry 272: 306–312. 10.1016/j.foodchem.2018.08.041. 30309548

[jfds70690-bib-0085] Zhu, J. , S. Zhang , Y. Liu , S. Chen , and L. Li . 2022. “Modelling and Assessment of Plasticizer Migration and Structure Changes in Hydrophobic Starch‐Based Films.” International Journal of Biological Macromolecules 195: 41–48. 10.1016/j.ijbiomac.2021.11.138. 34838859

